# Virological COVID-19 surveillance in Bavaria, Germany suggests no SARS-CoV-2 spread prior to the first German case in January 2020

**DOI:** 10.1007/s15010-021-01611-y

**Published:** 2021-04-23

**Authors:** Ute Eberle, Susanne Heinzinger, Regina Konrad, Clara Wimmer, Bernhard Liebl, Katharina Katz, Nikolaus Ackermann, Andreas Sing, Bavarian SARS-CoV-Public Health Laboratory and Epidemiology Team, Katja Bengs, Anja Berger, Stefanie Böhm, Merle Böhmer, Alexandra Dangel, Volker Fingerle, Sabrina Hepner, Bernhard Hobmaier, Martin Hoch, Gaia Lupoli, Gabriele Margos, Durdica V. Marosevic, Mercy Okeyo Bianca Treis, Tom Woudenberg

**Affiliations:** 1 Unit of Virology, Bavarian Health and Food Safety Authority, Oberschleißheim, Germany; 2 Unit of Public Health Microbiology, Bavarian Health and Food Safety Authority, Bayerisches Landesamt Für Gesundheit Und Lebensmittelsicherheit (LGL), Veterinärstraße 2, 85764 Oberschleißheim, Germany; 3 State Institute of Health, Bavarian Health and Food Safety Authority, Oberschleißheim, Germany; 4 Ludwig Maximilians-Universität, Munich, Germany; 5 Unit of Infectious Diseases Epidemiology, Bavarian Health and Food Safety Authority, Oberschleißheim, Germany

**Keywords:** Influenza, COVID-19, SARS-CoV-2, RT-PCR, Surveillance

## Abstract

The Bavarian Influenza Sentinel (BIS) monitors the annual influenza season by combining virological and epidemiological data. The 2019/2020 influenza season overlapped with the beginning COVID-19 pandemic thus allowing to investigate whether there was an unnoticed spread of SARS-CoV-2 among outpatients with acute respiratory infections in the community prior to the first COVID-19 cluster in Bavaria. Therefore, we retrospectively analysed oropharyngeal swabs obtained in BIS between calendar week (CW) 39 in 2019 and CW 14 in 2020 for the presence of SARS-CoV-2 RNA by RT-PCR. 610 of all 1376 BIS swabs-contained sufficient material to test for SARS-CoV-2, among them 260 oropharyngeal swabs which were collected prior to the first notified German COVID-19 case in CW 04/2020. In none of these swabs SARS-CoV-2 RNA was detected suggesting no SARS-CoV-2 spread prior to late January 2020 in Bavaria.

## Introduction

The COVID-19 outbreak in Wuhan/China at the end of December 2019 led to a rapid spread of SARS-CoV-2 throughout the world [1–3]. In calendar week (CW) 5, 2020 (Jan 27, 2020), the Bavarian Health and Food Safety Authority (LGL), was informed about the first human SARS-CoV-2 infection in Germany [4–7] leading to a cluster of several infected persons which could be contained by an immediate public health response [4, 5]. The first COVID-19 cases within the WHO European Region were reported in France on January 24, 2020 with the onset of symptoms in the first patient on January 17 [6]. On March 11, 2020 WHO declared COVID-19 a pandemic [1, 2, 4].

Due to the lack of sufficient RT-PCR methods for SARS-CoV-2 detection in the early period of the pandemic, mainly symptomatic patients were tested and subsequently identified. The increasing availability of RT-PCR tests allowed to address issues regarding the time of origin and early spread of SARS-CoV-2 which were triggered by both public and scientific interests: (i) mainly at the beginning of the pandemic, misinformation claiming an already long-standing spread of SARS-CoV-2 months or even years before the end of 2019 was disseminated, partly with the aim to raise doubt about the necessity of public health measures towards a supposedly pre-existing pathogen [7]; (ii) phylogenetic analysis of genomic data estimated the start of the COVID-19 pandemic in the period of October 6 to December 11, 2019 [8], dating back several weeks before the first clinical human cases were detected in Wuhan/China; (iii) retrospective testing of respiratory material from intensive care influenza-like illness (ILI) patients in a hospital from the Paris area identified a COVID-19 patient becoming symptomatic on December 27, 2019 nearly three weeks before the first reported COVID-19 case in France [9]; (iv) environmental monitoring studies finding SARS-CoV-2 RNA in wastewater dating back to December 18, 2019 suggested SARS-CoV-2 circulation in Italy two months earlier than the first reported autochthonous Italian case [10].

During the influenza season 2019/2020, the Bavarian Influenza Sentinel (BIS) established in 2009 [11] was carried out to know the proportion of ILI among patients with acute respiratory infections (ARI) caused by influenza viruses and respiratory syncytial virus (RSV).

Prompted by the issues mentioned above we retrospectively analysed BIS oropharyngeal swabs from season 2019/2020 for the presence of SARS-CoV-2 RNA to test the hypothesis of cryptic SARS-CoV-2 spread among ARI outpatients in the community.

## Methods

### Specimen collection

BIS consists of approximately 75 general practitioners. On a weekly basis each practitioner took specimens (naso- or oropharyngeal swabs) from two randomly chosen ARI patients (one swab per patient) and sent them to the LGL for virological diagnosis [11].

### Laboratory diagnostics

Virus transport media from swabs obtained for lab-based influenza surveillance within BIS were immediately stored after initial influenza testing at -20 °C. For SARS-CoV-2 diagnosis RNA was extracted from stored samples as previously reported [12]. The following methods were used: RNAdvance Viral Kit on a Biomeki7 (Beckman Coulter, Indianapolis, USA), Mag-Bind Viral RNA XPRESS Kit (Omega Bio-Tek) on a Hamilton Microlab Starlet (Hamilton, Bonaduz, Switzerland) followed by the use of AmpliCube Coronavirus SARS-CoV-2 PCR Kit (Mikrogen, Neuried, Germany) on the Bio-Rad CFX96 Touch Real-Time PCR Detection System (Bio-Rad, Feldkirchen, Germany). The assay detects the E-Gene of B-lineage betacoronavirus in FAM and the Orf1a-Gene specific for SARS-CoV-2 in HEX [12]. The threshold was manually set within the exponential phase of the detection curve.

### Data analysis, graphics

Data were evaluated and graphics were created using Spyder 4.1.4. (Python 3.8).

## Results

**Fig. 1:**
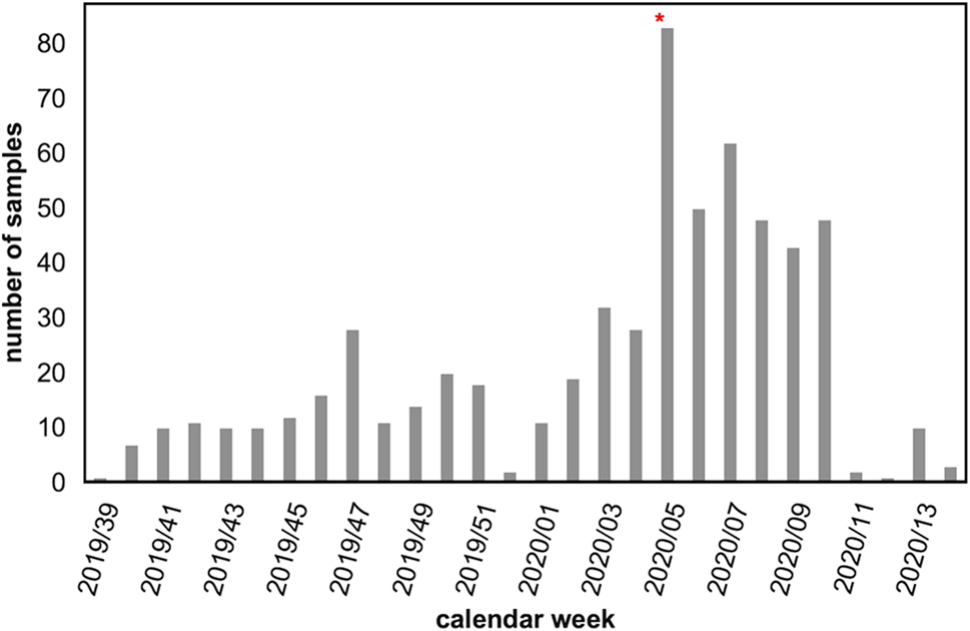
Number of samples analysed for SARS-CoV-2 by calendar week in the BIS 2019/2020 season. Grey bar: negative swabs, red star: first notified German COVID-19 case on 27.01.2020

From CW 39/2019 to CW 14/2020 1,376 specimens were analysed for influenza viruses. 610 of these samples for which sufficient material was available, were tested for SARS-CoV-2, among them 260 oropharyngeal swabs which were collected between CW 39/2019 and CW 04/2020 prior to the first notified German COVID-19 case. No SARS-CoV-2-positive results were obtained throughout the complete BIS 2019/2020 season (Fig. 1).

## Discussion

Some community-based sentinels or hospital-based patient surveys conducted outside Europe, e.g. Wuhan/China [13] and the US [14–16], retrospectively analysed respiratory samples mainly from ARI or ILI patients. The Wuhan [13] and Seattle/WA study sites [14] failed to identify SARS-CoV-2-RNA in 520 and approximately 400 samples, respectively, obtained prior to the first national confirmed COVID-19 case. Moreover, 11,400 samples from six US states prospectively collected within the month after the first verified US COVID-19 case did not render a SARS-CoV-2-positive result [15]. Similarly, a retrospective screening study on 1,700 nasopharyngeal samples from ILI patients in California covering the last two months of 2019 failed to reveal any SARS-CoV-2 positive patient [16].

European studies analysing respiratory samples from the period before a first national COVID-19 case was notified are even fewer. A French study on 14 intensive care ILI patients identified a COVID-19 patient retrospectively having become symptomatically at the end of December 2019, about three weeks prior to the first officially confirmed French case [9]. To the best of our knowledge, our study has retrospectively analysed the largest number of ARI patients from a community-based sentinel for SARS-CoV-2 infection by RT-PCR so far. In contrast to the French study [9], we could not identify a SARS-CoV-2-positive sample among 260 oropharyngeal swabs obtained between CW 39/2019 and 04/2020 prior to the first German case identified on January 27, 2020. Similarly, a retrospective analysis of 195 respiratory samples from the German CAPNETZ cohort of community-acquired pneumonia obtained during the 2019/2020 influenza season between December 2, 2019 (CW 48/2019) and April 28, 2020 (CW 17/2020) did not detect any SARS-CoV-2-infection before March 24, 2020 [17].

Limitations of our study are mainly due to its necessarily retrospective design relying on stored material. In addition, the BIS although comprising about 75 private medical physicians from all parts of Bavaria is not completely representative for the Bavarian population.

In conclusion, our study supports the hypothesis that there was no notable circulation of SARS-CoV-2 in the Bavarian population before the first German COVID-19 cluster at the end of January 2020.

## Data Availability

Data available within the article or its supplementary materials.
